# Prognostic significance of CNNM4 in ovarian cancer: a comprehensive bioinformatics analysis

**DOI:** 10.3389/fonc.2024.1483425

**Published:** 2024-12-03

**Authors:** Yiya Wang

**Affiliations:** School of Life Sciences, Qilu Normal University, Jinan, China

**Keywords:** CNNM4, ovarian cancer, prognosis, proliferation, migration

## Abstract

**Background:**

Ovarian cancer (OV) is a common malignancy in the female reproductive system, characterized by poor prognosis and high recurrence rates. The discovery of dependable molecular markers is crucial for improving the timeliness of detection, diagnosis, and treatment, ultimately aiming to lower fatality rates. CNNM4 (cyclin and CBS domain divalent metal cation transport mediator 4), a member of the CNNM (Cyclin M) family, binds to PRL (prolactin) to regulate magnesium homeostasis and influence tumor cell proliferation. Although CNNM4 is implicated in various cancers, its role in OV remains unclear.

**Methods:**

*In vitro* experiments assessed CNNM4 expression and its impact on the proliferation and migration of OV cells. Comparisons of TCGA and GTEx data were used to identify correlations between clinical features and outcomes. The role of CNNM4 in OV was further explored through comprehensive bioinformatics analyses.

**Results:**

Elevated levels of CNNM4 expression were observed in OV cells and tissues, and were linked to a poor prognosis. CNNM4 could modulate the proliferation and migration of various OV cell lines, including IOSE-80, SKOV-3, and A2780. Through involvement in multiple signaling pathways, evidenced by GSVA and GSEA, CNNM4 was implicated in OV progression. CNNM4 positively regulated the infiltration level of Macrophages M2, T cells CD4 memory resting and NK cells resting, and had a negative regulation effect on NK cells activated and T cells gamma delta. Moreover, CNNM4 is related to drug sensitivity of OV. A prediction model based on CNNM4 expression and clinical symptoms was constructed to predict OV prognosis.

**Conclusion:**

CNNM4 may affect the progression of OV and is associated with a poor prognosis. It has potential as a biomarker for predicting survival and as a target for therapeutic interventions in OV patients.

## 1 Introduction

Globally, OV is the fifth leading cause of cancer mortality among women, exemplifying a highly lethal condition that surpasses other gynecologic cancers in terms of mortality and recurrence rates (1, 2). OV is rare in women under 40, primarily presenting as germ-cell tumors, whereas epithelial cancers, which constitute over 90% of cases, predominantly affect women over 40. The risk of developing these tumors increases with age, peaking in those aged 70 and above (3). According to the statistics, 90% of OV cases are epithelial (EOV), with 60% being high-grade serous carcinomas (HGSCs) and the remaining 40% comprising clear cell, mucinous, endometrioid, and low-grade serous carcinomas (4). Studies have predicted that the incidence of OV and the death toll will increase annually (5). Although early-stage OV has a high cure rate (6, 7), a significant proportion of women are diagnosed with stage III/IV disease, with more than 75% presenting with late-stage OV and succumbing to the disease (8). In the past, conventional treatment methods for OV in clinical practice have included cytoreductive surgery and combination platinum-taxane chemotherapy. With ongoing advancements in clinical treatment technologies, many emerging treatments, such as small molecule inhibitors, are increasingly being applied (9). However, the cure rate for OV has not improved over the last 30 years, due to ineffective early detection tests and frequent recurrences due to chemotherapy resistance (10, 11). Consequently, enhancing early detection is vital for reducing mortality in women, alongside the need to pinpoint more effective biomarkers for OV.

CNNM4 belongs to the CNNM family, which can control intracellular Mg^2+^ levels through Na^+^/Mg^2+^ exchange, its biological function is linked to various diseases (12, 13). Members of the CNNM family have a highly conserved domain and are evolutionary conserved (14). CNNM4 was the first family member shown to the Mg^2+^-transporting function. In HEK293 cells, CNNM4 can reduce Mg^2+^ levels and increase the Na^+^ levels (15). Dyshomeostasis of magnesium, often found in cancer, contributes to pathophysiology. Adequate magnesium is essential for cell proliferation (16), and overexpression of Mg^2+^ channels has been implicated in tumor development and progression. PRL, frequently overexpressed in cancers, acts as a pseudo phosphatase by binding to CNNM4, modulating magnesium homeostasis. The co-expression of PRL with CNNM4 inhibits CNNM4-mediated Mg^2+^ efflux (14). Changes in intracellular magnesium, linked to cancer progression, result from the formation of a PRL-CNNM complex. In certain digestive system cancers, CNNM4 mRNA levels are elevated compared to normal tissues. High CNNM4 mRNA expression correlates with reduced overall survival in patients with pancreatic adenocarcinoma (17). These findings suggest CNNM4 may have regulatory roles in cancer pathogenesis and progression beyond its Mg^2+^ transport function, warranting further investigation into specific mechanisms. CNNM4 also plays a crucial role in genetic diseases. In patients with Jalili syndrome, CNNM4 mutations have been observed (18). And the presence of a CNNM4 defect has the potential to impact the individual’s fertility. The most common phenotype in CNNM4-deficient mice is male sterility (19). Recent studies have linked CNNM4 to tumor growth and metastasis, sparking interest in its potential as a target for cancer therapy. However, the relative expression and function of CNNM4 in OV remain unclear and require further investigation.

This study explores CNNM4’s relationship with OV, examining its varied expression in diseased versus normal tissues and its association with the clinical characteristics of OV to determine its prognostic significance. We demonstrated that CNNM4 promotes cancer proliferation and metastasis through multiple biological assays. GSVA and GSEA were conducted to analyze the biological mechanisms modulated by CNNM4 involved in OV pathogenesis. Furthermore, we explored the relationships between CNNM4 levels, immune infiltration, and drug sensitivity. Additionally, OV cells were categorized into subtypes and CNNM4 expression at the single-cell level was examined. A model predicting OV outcomes was formulated, underscoring the association between heightened CNNM4 expression and adverse prognoses. Therefore, CNNM4 represents a promising molecular marker for the diagnosis and therapeutic targeting of OV.

## 2 Materials and methods

### 2.1 Data acquisition and difference analysis

Clinical and genomic data from OV patients in the TCGA Pan Cancer Atlas cohort were download through the NCI Genomics Data Commons Portal (https://portal.gdc.cancer.gov/). The total population of that cohort is 429 samples. In addition, normal ovarian samples were acquired through the GTEx (The Genotype-Tissue Expression) database (n = 88) (https://www.gtexportal.org/home/index.html) (20). After merging the datasets from TCGA and GTEx, adjustments were made to the calculations to assess gene expression variations among different cancer types, especially in terms of CNNM4 expression. Quality control was performed during merging to reach reliable data, and because of this, nine samples of low or incomplete quality were removed. Single cell data file GSE184880 was then downloaded from the NCBI (National Center of Biotechnology Information) GEO (Gene Expression Omnibus) database and added sample data on 7 cases with complete expression profiles for further analysis.

### 2.2 Functional verification of CNNM4

qRT-PCR and Western Blot (WB) were performed for the quantification of CNNM4 expression in OV cells. Immunohistochemistry (IHC) was done for locating the expression of CNNM4 in OV and paracancerous tissue. Vector pcDNA3.1-CNNM4 was constructed to upregulate the mRNA expression of CNNM4. The CCK8 (Cell Counting Kit-8) assay was adopted for proliferation assessment. Flow cytometry was conducted to assess cell cycle and apoptosis. A wound healing assay was conducted for assessing cell invasion and migration. Detailed materials and methods can be found in the **Supplementary Material** section.

### 2.3 Acquisition and analysis of DepMap gene loss of function screening data

The DepMap portal (https://depmap.org/) was used to obtain such gene loss-of-function screening data for all available OV cell lines, in order to determine functional impact of CNNM4 in OV cells. The DepMap platform uses CRISPR/Cas9 technology to achieve gene knockout and allows probing into the dependencies of genes across diverse cancer cell lines. In this study, genetic screening data of all OV cell lines were selected, focusing more precisely on the analysis of the effect of knockout of the CNNM4 gene concerning cell viability and proliferation. Data were obtained from the DepMap web interface, and rates of survival in cell culture following knockout of the CNNM4 gene were analyzed using standard statistical methods, with the results visualized as box plots (21, 22).

### 2.4 Co-expression analysis

In this study, the expression of the CNNM4 gene and its pattern of co-expression in OV datasets were investigated by setting a threshold correlation coefficient at 0.4, using a significance level of 0.05. Circular correlation diagrams and heatmaps depicting the relationships between CNNM4 and other genes remarkably expressed were drawn using “corrplot” and “circlize” packages.

### 2.5 Gene enrichment analysis

To assess changes in biological function, the Gene Set Variation Analysis (GSVA) algorithm was used to analyze gene sets from the Molecular Signatures Database (23). Gene Set Enrichment Analysis (GSEA) was used to identify signaling pathways that were differently activated in groups with high vs low expression of CNNM4 after stratifying OV patients by their expression levels (24). To compare gene expression across subtypes and analyze subtype pathways, the version 7.0 of the MsigDB (The Molecular Signatures Database) database were downloaded and used as the background gene set (23, 25–27). Priority was given to enriched gene sets with an adjusted p-value < 0.05.

### 2.6 Immune cell infiltration analysis

Using the CIBERSORT method, we looked for relationships between gene expression patterns and immune cell compositions in OV samples, and we estimated the relative abundances of 21 different types of immune infiltrating cells.

### 2.7 Drug sensitivity analysis

Using the comprehensive GDSC (Genomics of Drug Sensitivity in Cancer) database, (https://www.cancerrxgene.org/) (28), the R package “pRRophetic” was used to forecast the sensitivity of individual tumor samples to chemotherapy. We employed regression analysis to establish the half maximum inhibitory concentration (IC50) for each chemotherapeutic therapy. To ensure the accuracy of regression and prediction, we used the GDSC training set for 10-fold cross-validation. Default settings such as “combat” were applied for batch effect removal, and averaging duplicate gene expressions was employed.

### 2.8 TMB, MSI, NEO data analysis

We operationalized Tumor Mutation Burden (TMB) as mutation frequency and variant number per exon length in individual tumor samples, further calculating the ratio between nonsynonymous mutation sites and total length of protein-coding regions (29). The microsatellite instability (MSI) value for each patient in the TCGA cohort was downloaded from a previous study (30). Neo-antigen assessment of each patient was calculated by NetMHCpanv3.0 (31).

### 2.9 Single cell sequencing analysis

Data processing was performed using the Seurat package, and spatial relationships between clusters were defined using the tSNE algorithm. The celldex package was used for annotating cells involved with tumor development. Similarly, single cell expression profiles identified the marker genes for each cell subtype by setting logfc.threshold = 1 in the function FindAllMarkers.

### 2.10 Nomogram model construction

A nomogram was developed as a clinical tool to forecast the prognosis of OV patients using the rms package and the cph function, incorporating age, grade, and CNNM4 gene expression as predictors. This nomogram was constructed by applying the regression coefficients from the model to the nomogram function. Scores were assigned to the levels of each variable, with total scores calculated by summing these individual scores, thereby estimating the 1-year and 3-year survival probabilities. This nomogram provides a personalized survival prediction for patients by integrating multiple variables.

### 2.11 Statistical analysis

Statistical analyses were conducted utilizing R software (version 4.2.2). The differential expression of the CNNM4 between normal and OV tissues was assessed through the Wilcoxon test. Additionally, logistic regression and the Wilcoxon test analyzed the relationship between CNNM4 expression level and various clinicopathological characteristics such as age, survival status, and tumor grade. The influence of CNNM4 and other clinical determinants on prognosis was explored via Cox regression and Kaplan-Meier survival analysis. All statistical assessments were performed using SPSS (Chicago, IL, USA), setting the significance threshold at P < 0.05. “*” indicates P < 0.05, “**” indicates P < 0.01, “***” indicates P < 0.001, “****” indicates P < 0.0001.

## 3 Results

### 3.1 Elevated CNNM4 expression and survival analysis in OV

**Figure 1:**
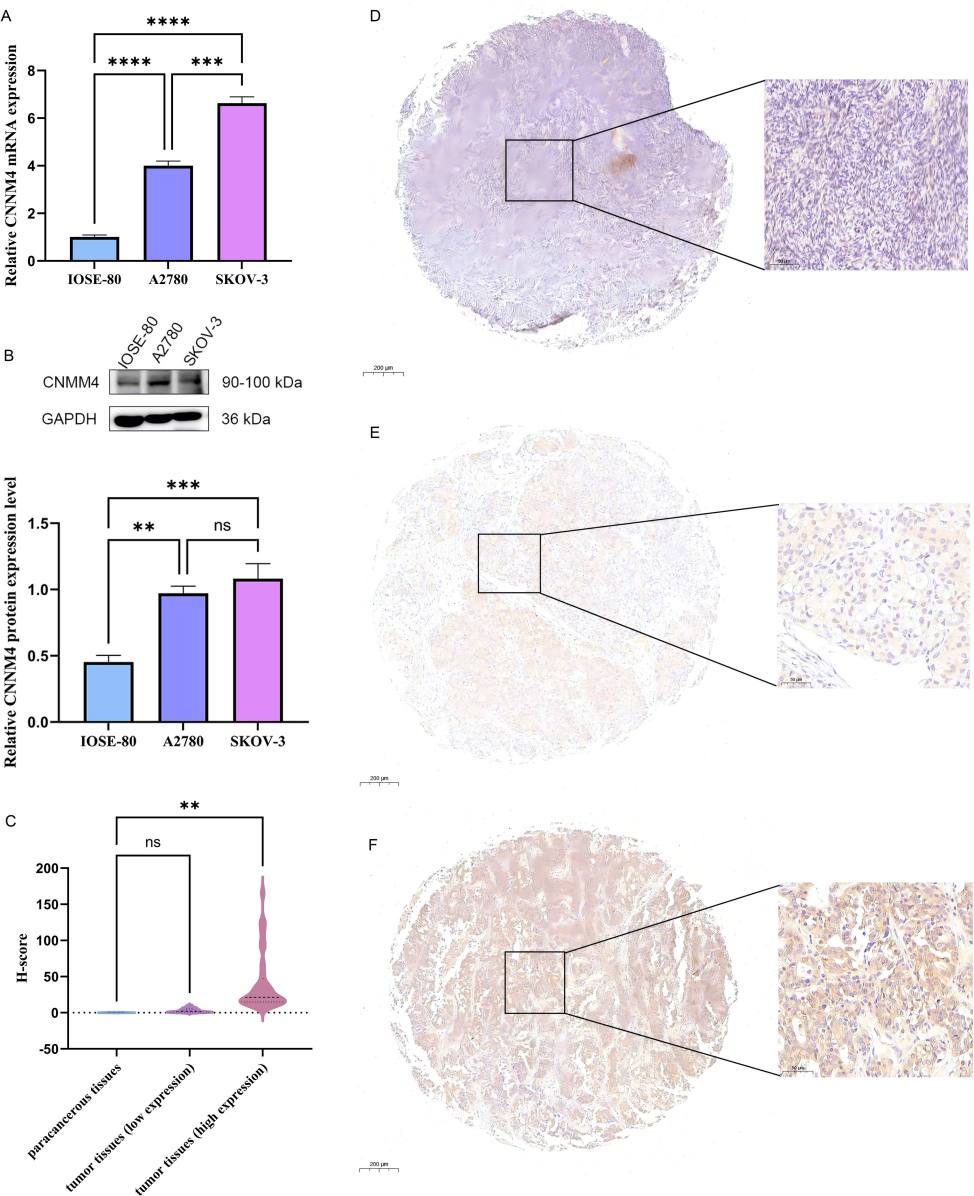
Elevated expression of CNNM4 in OV cells and tissues. **(A)** qRT-PCR and **(B)** WB analysis was performed to quantify CNNM4 expression in IOSE-80, A2780, and SKOV-3 cells, normalized against GAPDH expression. **(C)** Comparisons of relative CNNM4 protein expression in tumor tissues versus paracancerous tissues using H-scores. Immunoexpression of CNNM4 protein in paracancerous tissue **(D)**, OV tumor tissues (low expression) **(E)** and OV tumor tissues (high expression) **(F)** (× 200 magnification). “ns” indicates P > 0.05, “**” indicates P < 0.01, “***” indicates P < 0.001, “****” indicates P < 0.0001.

**Figure 2:**
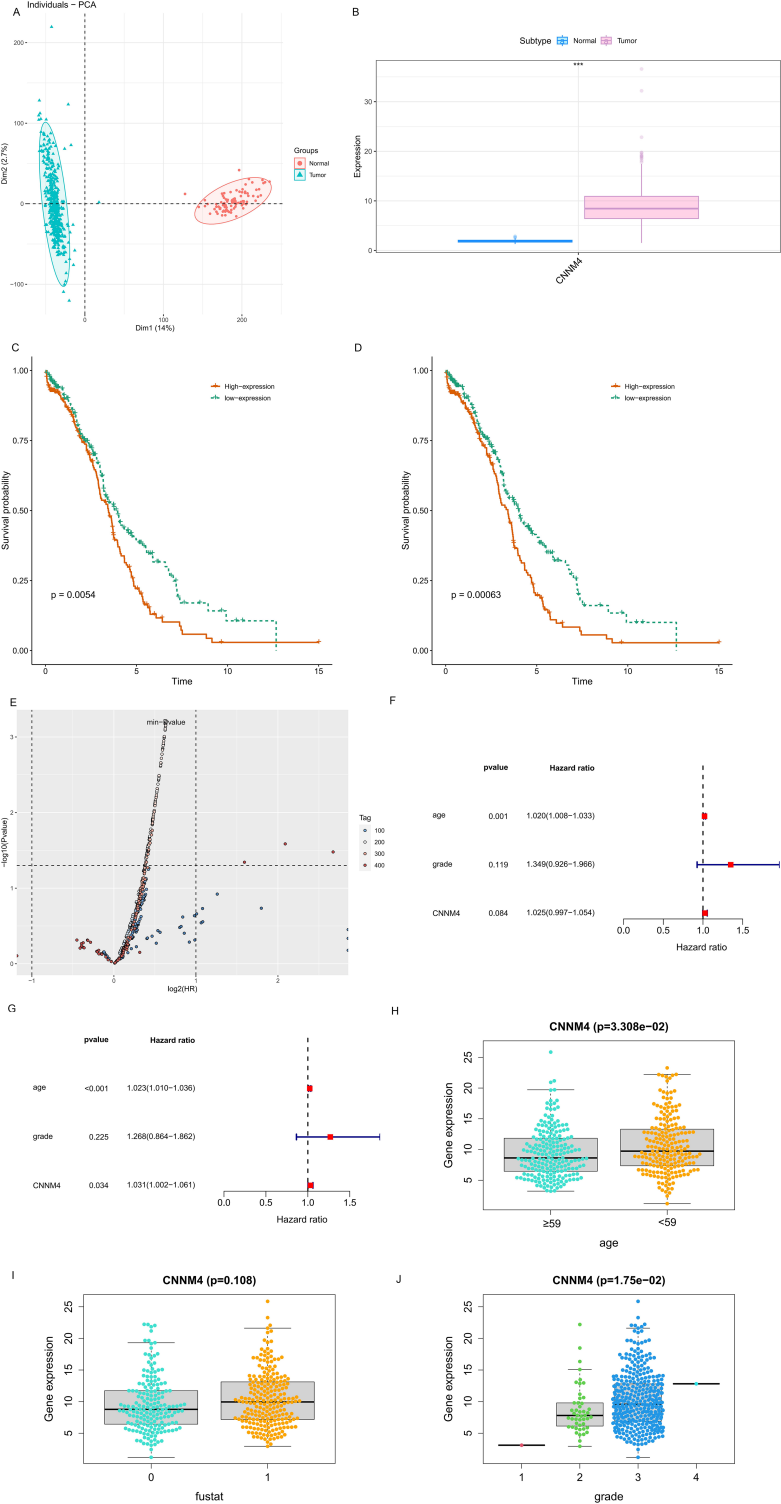
Elevated CNNM4 expression and survival analysis in OV. **(A)** Component analysis flash point diagram. **(B)** Variations in CNNM4 gene expression between control (blue) and tumor (pink) groups **(C)** Disparity in survival probabilities using median value as the cutoff. **(D)** Discrepancy in survival probabilities with the optimal value as the cutoff. **(E)** P value volcano plot. Single-factor **(F)** and multi-factor **(G)** Cox prognostic factor analyses in OV. Clinical relevance of CNNM4 expression assessed in OV. **(H)** Age, **(I)** Fustat, **(J)** Grade.

Initially, CNNM4 expression levels were assessed in IOSE-80, SKOV-3, and A2780 cells using qRT-PCR and WB. A notable increase in CNNM4 expression level was observed in SKOV-3 and A2780 cells compared to IOSE-80 cells (**Figures 1A, B**). H-scores revealed significantly higher CNNM4 immunoexpression in tumor tissues (**Figure 1C**), and IHC revealed lower CNNM4 staining in the cytoplasm of paracancerous cells (**Figure 1D**) than in tumor cells (**Figures 1E, F**). Then a baseline table based on the data from TCGA was created according to the expression level of CNNM4 (**Table 1**). Integration with GTEx data (**Figure 2A**) demonstrated elevated CNNM4 levels in OV tissues (**Figure 2B**). The overall survival analysis revealed statistically significant differences in survival probability between two groups when utilizing the median value as the cut-off point (p=0.0054) (**Figure 2C**), persisting under optimal cutoff value (p=0.00063) (**Figures 2D, E**). Both single-factor and multi-factor Cox regression models identified age as a risk factor (**Figures 2F, G**), and a significant association was found between CNNM4 expression, patient age, and tumor grade in survival analysis (**Figures 2H–J**).

**Table 1:** Relationships between CNNM4 expression and clinical pathological features of patients with OV.

	High (N=210)	Low (N=210)	P-value
Fustat			
Alive	82 (39.0%)	104 (49.5%)	0.0391
Dead	128(61.0%)	106(50.5%)	
Age			
≥59	97(46.2%)	114(54.3%)	0.118
<59	113(53.8%)	96(45.7%)	
Grade			
G1	0(0%)	1(0.5%)	0.0215
G2	13(6.2%)	34(16.2%)	
G3	189(90.0%)	172(81.9%)	
G4	1(0.5%)	0(0%)	
GB	1(0.5%)	1(0.5%)	
GX	4(1.9%)	2(1.0%)	
unknow	2(1.0%)	0(0%)	

### 3.2 The Biological Function Analysis of CNNM4

**Figure 3:**
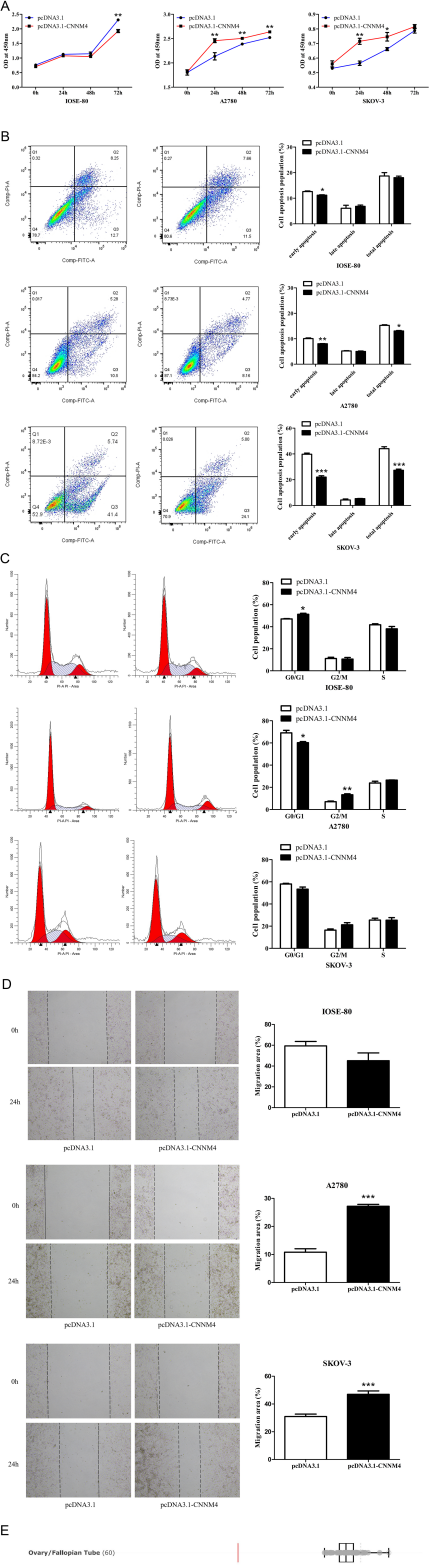
Impacts of CNNM4 on cellular growth and migration. **(A)** CCK8 assays to evaluate cell proliferation at 0, 24, 48, and 72 hours in cells overexpressing CNNM4. **(B)** Apoptosis in cells transfected with pcDNA3.1 and pcDNA3.1-CNNM4, analyzed via flow cytometry. **(C)** Cell cycle analysis depicted in flow cytometry images, presented as a percentage of total cells. **(D)** Migration assessed in cells transfected with pcDNA3.1 and pcDNA3.1-CNNM4 using wound healing assays, with images captured at 0 and 24 hours across three independent experiments. The data presented in this study are reported as the mean ± SEM. “*” indicates P < 0.05, “**” indicates P < 0.01, “***” indicates P < 0.001. **(E)** Gene loss-of-function screening results of CNNM4 gene in ovarian/fallopian tube (OV) cell lines.

To investigate the impact of CNNM4 on the progression of OV, the pcDNA3.1-CNNM4 vector was constructed to enhance CNNM4 expression. Following transfection, cells exhibited efficient overexpression of CNNM4 (**Supplementary Figure S1**). The proliferative capacity of the cells was assessed using the CCK8 assay at time points of 0, 24, 48, and 72 hours post-transfection. In IOSE-80 cells, viability was inhibited in the pcDNA3.1-CNNM4 group, whereas in A2780 and SKOV-3 cells, the results were opposite (**Figure 3A**). Flow cytometry was employed to evaluate apoptotic potential. In IOSE-80 cells, the percentage of early apoptotic cells was reduced in the pcDNA3.1-CNNM4 group, but there were no significant differences in the overall proportion of apoptotic cells between the pcDNA3.1 and pcDNA3.1-CNNM4 groups, indicating a minor effect of CNNM4 on apoptosis of IOSE-80 cells. In contrast, in A2780 and SKOV-3 cells, the proportion of total apoptotic cells significantly decreased in the pcDNA3.1-CNNM4 group, suggesting that CNNM4 overexpression reduced apoptotic capacity (**Figure 3B**). In cell cycle assays, IOSE-80 cells showed a higher G1 phase proportion in the pcDNA3.1-CNNM4 group, whereas the A2780 group displayed a reduction in G1 phase cells, with increases in S and G2/M phases. No notable changes were observed in the SKOV-3 cells (**Figure 3C**). Migration capabilities were assessed using a wound-healing assay. In A2780 and SKOV-3 cells, the pcDNA3.1-CNNM4 group exhibited a significantly reduced scratch wound distance (**Figure 3D**). These findings suggest that CNNM4 may impact cellular proliferation and migration. The influence of CNNM4 on OV cells was further analyzed using gene loss-of-function data from the DepMap portal. The results indicated that CNNM4 knockdown did not significantly reduce survival or inhibit proliferation in some OV cell lines (**Figure 3E**; **Supplementary Table S1**).

**Figure 4:**
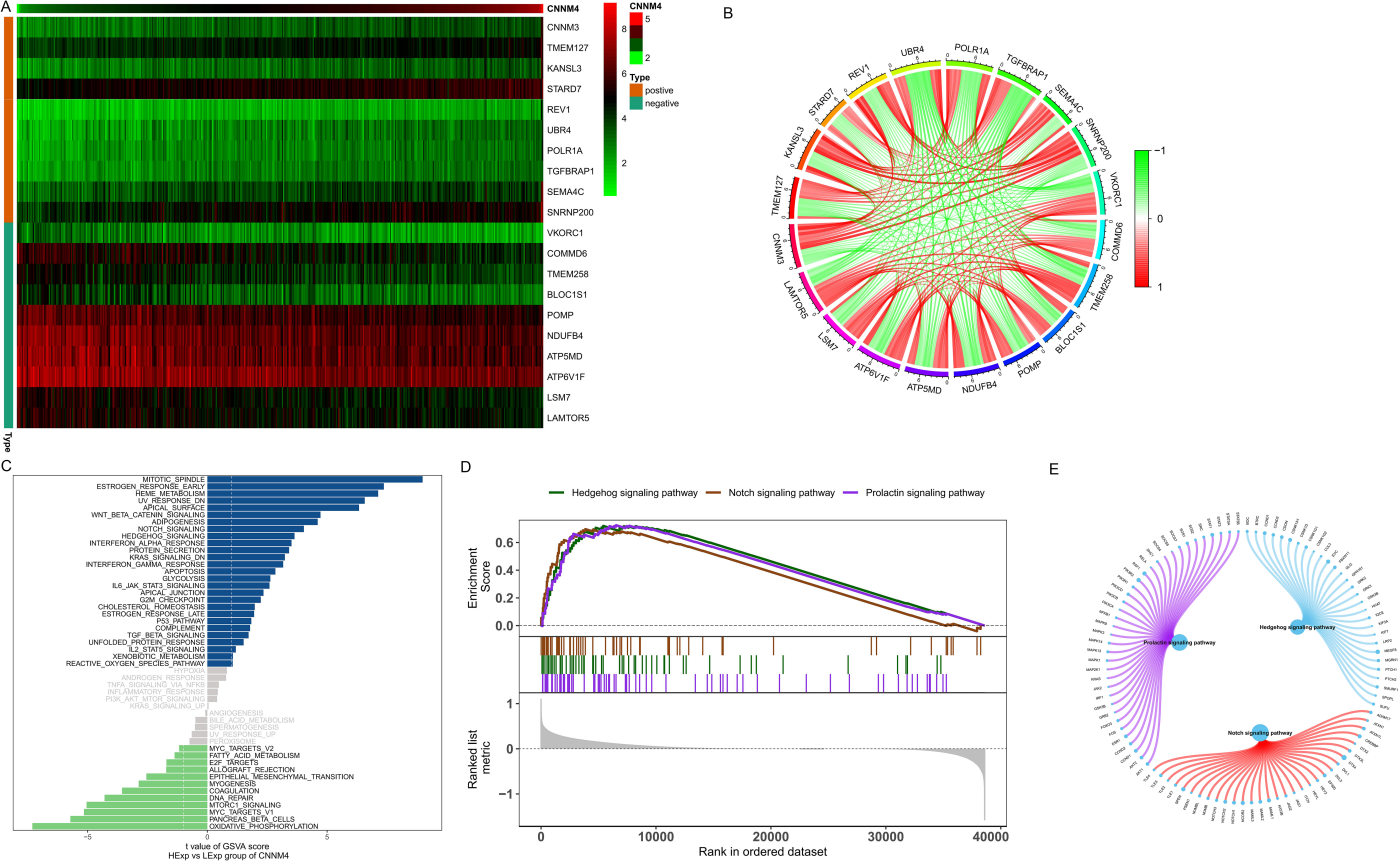
Biological function analysis of CNNM4. **(A)** Correlation coefficients showing positive and negative correlations among the top 10 genes. **(B)** Co-expression correlation circle diagram, red for positive and green for negative correlations. **(C)** GSVA utilized to evaluate pathway activities of key genes in high and low CNNM4 expression groups, identifying potential pathway disparities between the high-risk and low-risk groups. Hallmark sets served as the background gene set. **(D, E)** Analysis of KEGG signaling pathways and gene involvement in pathway regulation performed by GSEA.

To explore the potential biological functions of CNNM4 further, a correlation interaction network was established based on expression profiles from the TCGA database, with a correlation coefficient threshold of 0.4 and a significance level of 0.05. A total of 150 genes significantly associated with CNNM4 were identified, with the top 10 displayed in a heatmap (**Figure 4A**) and illustrated in a co-expression circle diagram (**Figure 4B**). Specific signaling pathways in which CNNM4 is involved were also examined. GSVA results indicated significant enrichment in pathways such as HEME_METABOLISM, KRAS_SIGNALING_DN, and IL6_JAK_STAT3_SIGNALING (**Figure 4C**). Additionally, GSEA revealed significant enrichment in the Hedgehog, Notch, and PRL signaling pathways (**Figures 4D, E**), suggesting that CNNM4 may influence OV progression through these pathways.

### 3.3 The relationship between CNNM4 expression and immune infiltration

**Figure 5:**
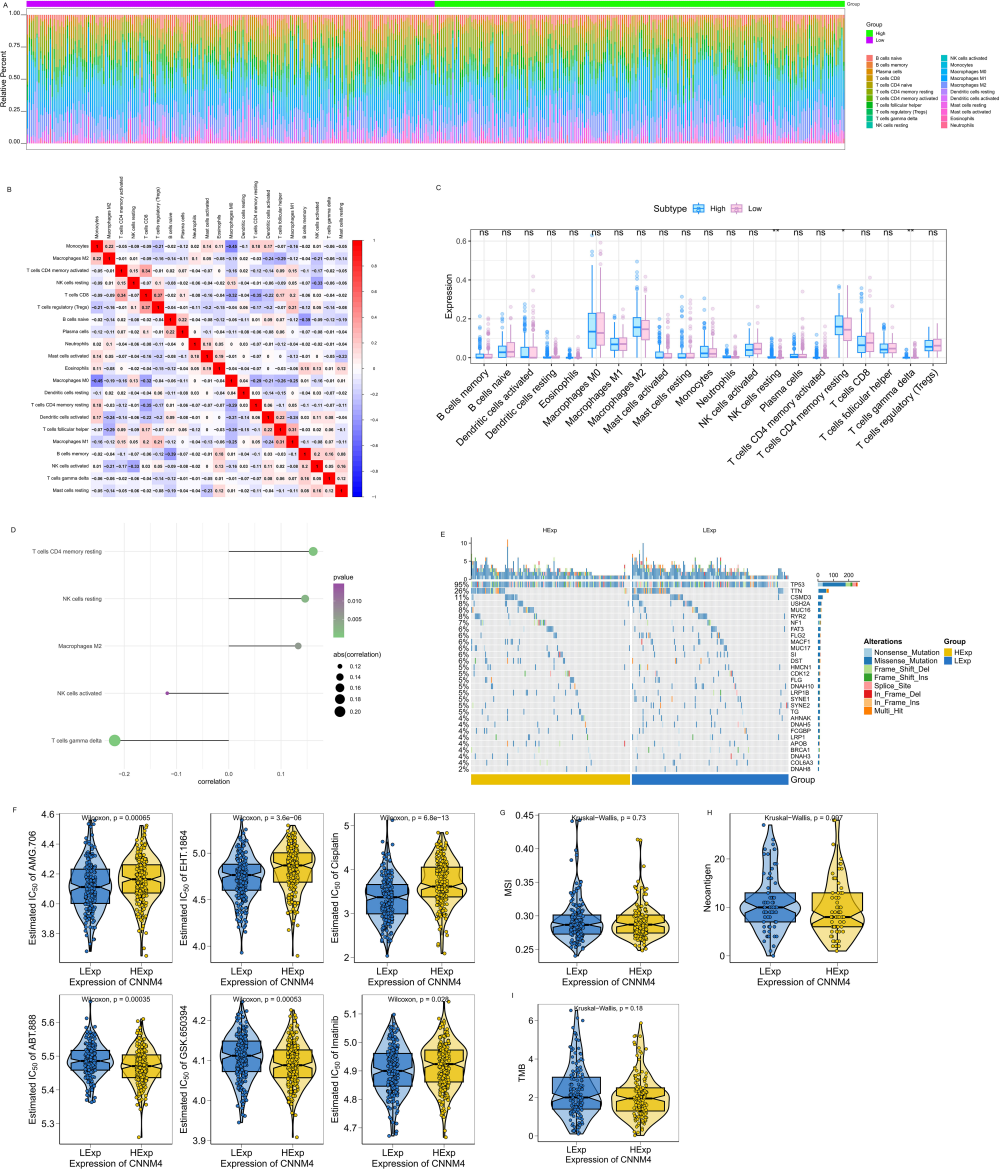
CNNM4 expression is correlated with immune infiltration, mutations, drug sensitivity, MSI, NEO and TMB. **(A)** Relative percentages of 22 immune cell subtypes. **(B)** Pearson correlations for 21 immune cell types, with blue for negative and red for positive correlations. **(C)** Differences in immune cell quantities between patients with high and low CNNM4 expressions, colored blue and pink, respectively. “ns” indicates P > 0.05, “*” indicates P < 0.05, “**” indicates P < 0.01. **(D)** Correlation between CNNM4 expression and immune cell content. **(E)** Analysis of the top 30 high-frequency mutated genes from SNP data in OV to explore differences between patient groups. **(F)** Prediction of potential therapeutic agents from the GDSC database data: AMG.706, EHT.1864, Cisplatin, ABT.888, GSK.650394, and Imatinib. **(G)** Correlation analysis between CNNM4 expression and MSI. **(H)** Correlation analysis between CNNM4 expression and NEO. **(I)** Correlation analysis between CNNM4 expression and TMB.

Additional studies were performed to clarify CNNM4’s role in OV. Immune cell proportions and their correlations in patients were analyzed (**Figures 5A, B**). After stratifying patients by median value of CNNM4, significant variations were noted among resting NK cells, CD4 memory resting T cells, and gamma delta T cells (**Figure 5C**). The potential molecular mechanisms by which CNNM4 affects OV progression were explored through analyses of the association between CNNM4 expression and tumor immune infiltration. Significant positive correlations were found between CNNM4 expression and T cells CD4 memory resting, NK cells resting, and Macrophages M2, as well as significant negative correlations with T cells gamma delta and NK cells activated (**Figure 5D**).

### 3.4 Map of the mutations associated with CNNM4

Processed single nucleotide polymorphism (SNP) data for OV were obtained and analyzed to identify disparities in mutated genes among patient cohorts. A mutation landscape was constructed using the R package Complex Heatmap, highlighting the top 30 genes with increased mutation rates, showing higher mutation frequencies in TP53 and other genes among patients with elevated CNNM4 expression (**Figure 5E**).

### 3.5 TMB, MSI, NEO and drug sensitivity

The correlation between CNNM4 expression and the sensitivity of common chemotherapy drugs was investigated using the R package “pRRophetic”. Significant associations were observed between CNNM4 expression and the sensitivity of several drugs, including AMG.706, EHT.1864, Cisplatin, ABT.888, GSK.650394, and Imatinib (**Figure 5F**). Additionally, the relationship between CNNM4 expression and well-known immunotherapy-related tumor markers was examined, revealing associations with MSI (**Figure 5G**), NEO (**Figure 5H**), and TMB (**Figure 5I**) across groups with varying levels of CNNM4 expression.

### 3.6 Analysis of CNNM4 expression in single cells

**Figure 6:**
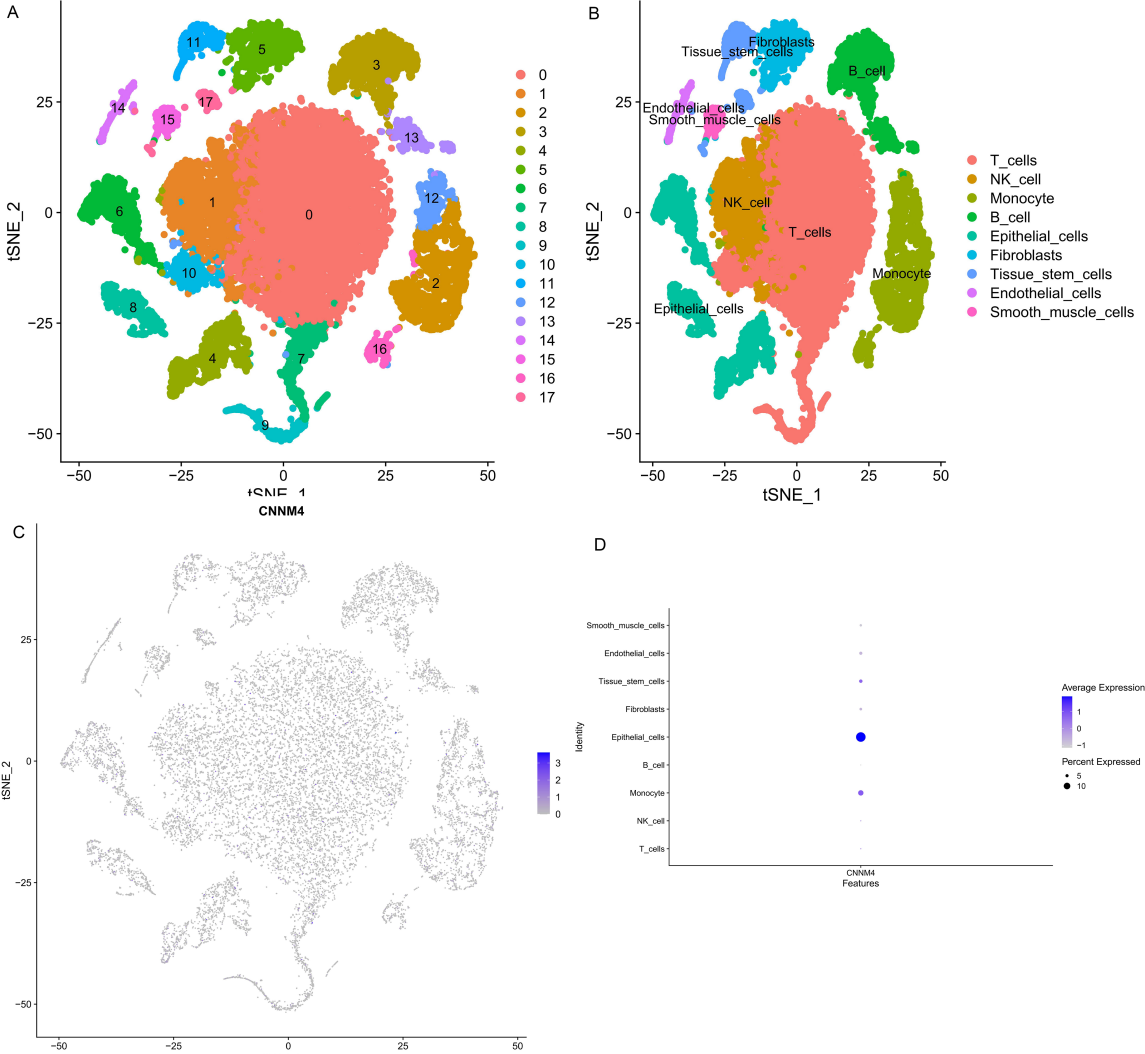
Single cell annotation. **(A)** Division of cells into 18 clusters via tSNE algorithm based on significant PCA components. **(B)** Annotations of 18 clusters identifying 9 as T cells, NK cell, Monocyte, B cell, Epithelial cells, Fibroblasts, Tissue stem cells, Endothelial cells, Smooth muscle cells in the 9 cell categories. **(C)** CNNM4 expression flash point map in cells. **(D)** Overview of CNNM4 expression in cells.

Single-cell transcriptomics data from GSE184880 were analyzed using Seurat package. Employing the tSNE algorithm, 18 cell subtypes were identified (**Figure 6A**), and these were further categorized into nine groups using SingleR: T cells, NK cells, monocytes, B cells, epithelial cells, fibroblasts, tissue stem cells, endothelial cells, and smooth muscle cells (**Figure 6B**). CNNM4 expression across these cell types was also investigated (**Figure 6C, D**).

### 3.7 Prediction analysis of CNNM4 expression and clinical indicators

**Figure 7:**
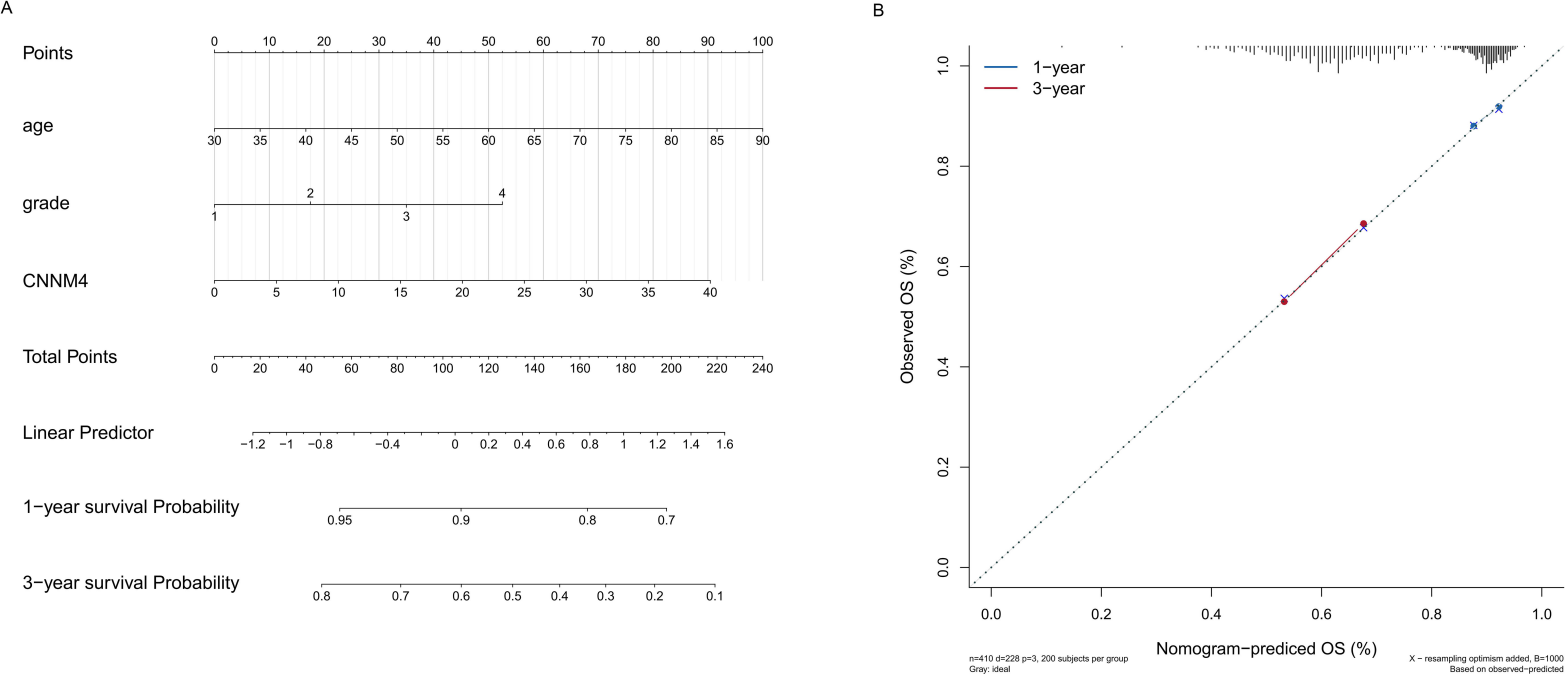
Establishment and validation of the prognostic nomogram. **(A)** Construction of a nomogram incorporating the CNNM4 signature and clinical data to forecast 1- and 3-year overall survival for OV patients in the TCGA dataset. **(B)** Utilization of calibration curves to verify the agreement between predicted and actual 1- and 3-year outcomes.

Given the results suggesting a role for CNNM4 in OV development, a nomogram was created integrating age, grade, and CNNM4 expression to predict 1- and 3-year overall survival (OS) outcomes (**Figure 7A**). The predictive accuracy of the nomogram was validated, showing close alignment between predicted and observed OS, thus confirming the model’s robust predictive capability (**Figure 7B**).

## 4 Discussion

Mg is a crucial cation in cellular environments, essential for various biological functions through interactions with intracellular molecules including lipids, proteins, and nucleotides (32). Disruptions in Mg homeostasis are implicated in the pathophysiology of numerous diseases, including cancer, although the connection between Mg and cancer initiation remains debated. There is substantial evidence indicating an inverse relationship between Mg intake and cancer development, a relationship that becomes more complex in advanced tumor stages. The necessity of Mg for cell proliferation suggests that rapidly dividing tumor cells are particularly dependent on Mg uptake, with studies confirming elevated intracellular Mg levels in such cells (16). CNNM4, part of the CNNM family, was initially discovered as a membrane protein with domains conserved across bacterial species, highlighting its fundamental biological role (33). CNNM4 was the first identified member of this family with Mg^2+^-transporting capabilities (15), and its interaction with PRL is known to regulate Mg homeostasis (14). The role of CNNM4 in cancer may be linked to its association with PRL, which promotes Mg^2+^ accumulation intracellularly, thereby supporting tumor growth and metastasis. High CNNM4 expression is observed in various cancers, where it correlates with poor prognosis due to its impact on cellular growth and immune modulation (17). Current research indicates that CNNM4 is significantly upregulated in OV and that its overexpression correlates with poor prognosis, likely due to its effects on cell growth and immune responses. This suggests that CNNM4 expression may serve as a reliable predictive marker for OV.

We conducted CNNM4 expression analysis in OV tissues and cells, and found that the levels of CNNM4 are significantly higher in tissues and cells of OV compared to paracancerous tissues and normal cells. The same trend has been reported in various tumors, such as esophageal carcinoma and adenocarcinoma of the pancreas, stomach, colon, and rectum (17). Thus, CNNM4 is assumed to be critical for tumor development. Moreover, the overexpression of CNNM4 was associated with poor prognosis. Though there are different points of view, many researchers have indicated that CNNM4 may serve as a predictive marker for cancers. The downregulation of CNNM4 has been detected by IHC in colorectal cancer tissues, showing an inverse correlation with malignancy grade of tumors (14). Further survival analysis conducted showed that higher CNNM4 expression was strongly associated with reduced OS, similarly observed in cases of pancreatic adenocarcinoma (17). This would hence mean that the role of CNNM4 is different in some cancers and requires further investigation. Additionally, patient age and histological grade were identified as independent prognostic factors for OV, hence further confirming the potential of CNNM4 as a prognostic marker.

Mg^2+^ has been long recognized for its critical role in regulating cell proliferation (34). CNNM4 is known to influence cellular Mg^2+^ concentrations (15). Disruption of magnesium homeostasis is linked to various diseases, including cancer. In this study, we analyzed CNNM4 expression in IOSE-80, A2780, and SKOV-3 cell lines, exploring its effects on cellular proliferation and migration. Our analysis showed elevated levels of CNNM4 in A2780 and SKOV-3 cells compared to IOSE-80 cells, indicating a possible role in facilitating OV progression. We also examined the effect of CNNM4 on cell proliferation and apoptosis, observing that CNNM4 inhibits proliferation and migration in IOSE-80 cells while showing opposite effects in A2780 and SKOV-3 cells. Recent research revealed that deficiency in CNNM4 boosts proliferation in mouse colonic epithelial cells, leading to an increase in polyp formation and the presence of invasive cancer cells in these mice, suggesting that CNNM4 disruption promotes tumor development and malignancy, thereby characterizing it as a tumor suppressor (14, 35). These different results may be due to the operational mechanisms of CNNM4 in OV may differ from the PRL-CNNM4 regulatory pathway. Further detailed studies are required to clarify the role of CNNM4 as an oncogene and the mechanisms through which Mg^2+^ dysregulation contributes to cancer progression in OV. The loss-of-function screening data showed that CNNM4 gene loss did not significantly affect survival rates in OV cell lines, nor did it significantly inhibit proliferation across some cell lines. These outcomes vary from those obtained in CCK8 assays, potentially due to differences in cell type, knockout efficiency, or experimental conditions. Although DepMap data indicates a minimal dependency on CNNM4 in certain OV cell lines, our *in vitro* findings provide direct evidence that CNNM4 overexpression in specific cell lines indeed enhances cell proliferation, supporting its pro-oncogenic role in OV.

In the analysis of CNNM4’s biological roles in OV, GSVA identified associations of CNNM4 expression with pathways such as HEME_METABOLISM, KRAS_SIGNALING_DN, and IL6_JAK_STAT3_SIGNALING. Heme is crucial for gas transport, oxidative metabolism, and detoxification processes (36). Previous studies have indicated that HEME_METABOLISM is associated with the colon cancer and invasion of OV cells (37, 38). Moreover, KRAS_SIGNALING_DN has been associated with gastric and glioblastoma cancers (39, 40), and IL6_JAK_STAT3_SIGNALING is implicated in the progression of gliomas, bladder, and prostate cancers (41–43). Moreover, GSEA revealed significant enrichment for Hedgehog, Notch, and PRL signaling pathways. These pathways affect tumorigenesis, development, and malignant phenotype in many cancers. Deregulation of the Hedgehog signaling pathway is associated with several cancer, such as basal cell carcinoma, medulloblastoma, breast, pancreatic, ovarian, colon and small-cell lung carcinomas (44). PRL plays a significant role in certain cancers, including OV (45, 46), and the co-expression of PRL with CNNM inhibits CNNM-mediated Mg^2+^ efflux, affecting intracellular magnesium levels linked to cancer progression (14). Notch signaling pathway has been implicated in the development and homeostasis of tissues and organs, the deregulation of which results in diseases or cancers. It would appear that recent data support the idea of the Notch signaling pathway playing a dual role in tumor promotion and inhibition (47). Notch signaling pathway could modulate immune cells which involved in anti- or pro-tumor responses, and this pathway also could be a potential target for cancer immunotherapy (48). These findings indicate that CNNM4 may promote OV progression by engaging multiple interacting molecular pathways. The occurrence and development process of tumor is very complex, and there are many signaling pathways and cytokines involved in it, and the specific situation needs to be confirmed by further research.

The tumor microenvironment, a complex environment comprising immune cells, nutrients, chemokines, and various other components (49), significantly influences tumor growth, invasion, metastasis, and chemoresistance (50, 51). Immunotherapy, which leverages this microenvironment to activate the immune system against tumor cells, underscores the importance of understanding the role of immune cells within this context to identify new immunotherapeutic targets. However, the specific interactions between CNNM4 and immune cell infiltration in OV remain unclear. In this study, we investigated the correlation between CNNM4 expression levels and 21 immune cell types in OV, revealing a significant relationship between CNNM4 expression and the content of NK cells resting, T cells CD4 memory resting, and T cells gamma delta. A comprehensive analysis revealed that CNNM4 exhibited a positive regulatory impact on the infiltration level of T cells CD4 memory resting, NK cells resting and Macrophages M2 while exerting a negative regulatory influence on T cells gamma delta and NK cells activated. These results underscore the significant role of CNNM4 in the tumor microenvironment and its influence on OV prognosis. Immune cells are critical in both promoting and inhibiting tumors; they can destroy cancer cells and prevent infections, yet tumors may evade immune detection (52). Particularly, T cells CD4 memory resting are crucial in the antitumor immune response (53). Alterations in NK cell resting and T cells CD4 memory resting populations have been observed in the tumor microenvironments of colon and bladder cancer patients (54, 55). Furthermore, Macrophages M2 are involved in the alternative activation of the Th2 cell response and can secrete factors like IL-10, TGF-β, PGE2, and VEGF that promote tumorigenesis and angiogenesis (56, 57). T cells gamma delta play a role in associations of various cancers, such as OV, hepatocellular carcinoma and prostate cancer (58–60). There is still a lot of uncertainty regarding the molecular mechanism of the relationship between CNNM4 and immune cells in OV, this is worthy to further exploration. The study findings indicated that patients categorized as high-risk exhibited poor prognosis in comparison to those in the low-risk group. The difference in outcomes may be attributed to the presence of an immunosuppressive microenvironment in the high-risk group. This could be attributed to the repressive microenvironment interfering with the normal functioning of tumor cytotoxic cells, hence promoting cancer progression and increasing the mortality rate in patients (61). Such an immunosuppressive state, if addressed through targeted therapies, will provide a better treatment modality for OV patients. Recently, chemotherapy has remained a mainstay treatment for OV cases. However, chemoresistance is likely to constitute the main determinant of therapeutic failures in most patients. Our drug sensitivity analysis identified that CNNM4 expression correlates with the responsiveness against several anticancer drugs, including AMG.706, EHT.1864, Cisplatin, ABT. 888, GSK.650394, and Imatinib. This could reflect that higher CNNM4 expression might be an indicator of drug sensitivity as well as resistance and thus can be regarded as a potential biomarker in the prediction of chemotherapeutic outcome in OV cells.

In conclusion, elevated CNNM4 expression in OV is associated with advanced histological grades and poor prognosis, affecting cellular proliferation and migration. The upregulation of CNNM4 influences multiple signaling pathways and correlates with changes in immune cell infiltration, underscoring its importance as a biomarker for diagnosing and predicting OV outcomes. Furthermore, the relationship between CNNM4 expression and the sensitivity of OV cells to antitumor treatments highlights its potential utility in therapeutic strategies. These findings collectively emphasize the value of CNNM4 as a biomarker for OV diagnosis and prognosis.

## Data Availability

The raw data supporting the conclusions of this article will be made available by the authors, without undue reservation.
