# Supplementary material for: Prognostic significance of CNNM4 in ovarian cancer: a comprehensive bioinformatics analysis
Source: Front Oncol. 2024 Dec 3;14:1483425. doi: 10.3389/fonc.2024.1483425 (PMC11649545; doi:10.3389/fonc.2024.1483425)
Supplement: Supplementary file 2 [file DataSheet2.docx]

Supplementary Material

# **Cell culture**

The IOSE-80, A2780, and SKOV-3 cell lines were selected for the purpose of detecting CNNM4 expression levels and were utilized in the in vitro gain-of-function investigation. The cell lines utilized in this study were obtained from iCell Bioscience, Shanghai, China. The IOSE-80 and SKOV-3 cell lines were grown in RPMI 1640 medium (HyClone) containing 10% fetal bovine serum (FBS) and 1% penicillin/streptomycin. The A2780 cell line was cultured in DMEM supplemented with 10% FBS and 1% penicillin/streptomycin. The cell cultures were subjected to incubation at 37 °C in an atmosphere that was humidified with 5% CO_2_.

# **Western Blot**

The total protein was isolated from cells using Cell lysis buffer for Western and IP (Beyotime) and the protein concentration was determined using the Enhanced BCA Protein Assay Kit (Beyotime). The CNNM4 polyclonal antibody was purchased from Proteintech, Inc. The GAPDH monoclonal antibody was purchased from Beyotime, Inc. Equal amounts of protein were separated by 8% sodium dodecyl sulfate gel electrophoresis under denaturing and non-reducing conditions and then transferred to polyvinylidene fluoride (PVDF) membranes. The PVDF membranes were blocked, then incubated (1 h, 37°C) with the CNNM4 antibody (dilution, 1:5,000; 14066-1-AP; Proteintech, Wuhan, China) and GAPDH antibody (dilution, 1:1,000; AF1186; Beyotime; Shanghai, China) which diluted in Primary Antibody Dilution Buffer (Beyotime). After washing in phosphate-buffered saline with Tween 20, the blots were incubated with horseradishperoxidase-conjugated goat anti-rabbit immunoglobulin G antibody (dilution, 1:2,000; A0208; Beyotime; Shanghai, China) which diluted in Secondary Antibody Dilution Buffer (Beyotime). The signals were visualized using a BeyoECL Star kit (Beyotime).

# **Immunohistochemical Staining**

Tissue microarray (TMA) (Cat No. YP-FOV8011a) paraffin blocks of OV tissues were purchased from Shanghai Yblbio (Shanghai, China). A total of 80 pairs of cancerous and paracancerous tissue samples were subjected to IHC staining. Each TMA slide was first stained with a rabbit anti-CNNM4 antibody (dilution, 1:1,000; 14066-1-AP; Proteintech, Wuhan, China) and then incubated with horseradish peroxidase-conjugated goat anti-rabbit immunoglobulin G antibody (dilution, 1:50; A0208; Beyotime; Shanghai, China). After rinsing, color was developed using 3, 3′-diaminobenzidine (DAB, Servicebio, Inc.). Sections were counterstained with hematoxylin and photographed at 400×magnification using an XSP-C204 microscope (COIC, Chongqing, China). Images were then captured using Pannoramic viewer (3DHISTECH Kft; Budapest, Hungary) and analyzed using HALO. Immunohistochemistry score (H-SCORE) was calculated as H-SCORE=∑(PI × I) = (percentage of cells with weak intensity × 1) + (percentage of cells with moderate intensity× 2) + (percentage of cells with strong intensity × 3), where PI is the proportion of positive cells among all cells in the section and I is the coloration intensity. Cancerous tissue samples were divided into low and high expression groups according to H-score, and compared with paracancerous tissue samples, respectively.

# **Transfection**

Upon reaching a cellular confluence of 70%, the cells underwent transfection with pcDNA3.1 vector and pcDNA3.1-CNNM4 vector utilizing Lipofectamine 2000 reagent (Invitrogen). The cells were utilized for subsequent experiments 48 h post-transfection.

# **RNA extraction and qRT-PCR**

The extraction of total RNA from cell lines was performed by utilizing the TRIzol reagent (Invitrogen, Carlsbad, CA, USA) following the directions provided by the manufacturer. The reverse transcription of total RNA (1μg) was conducted employing a FastKing gDNA Dispelling RT SuperMix (Tiangen, Beijing, China). The qPCR procedure was conducted employing Talent qPCR PreMix (Tiangen, Beijing, China) following the manufacturer's rules. The design of CNNM4 primer was performed in the following manner: forward: 5’-TAGACAACCTCATCGGGTC-3', reverse: 5’-AACAGTGCGAATCTCCTG-3'. GAPDH served as set an internal reference (forward: 5'-CTGCACCACCAACTGCTTAG-3’, reverse: 5’-GTCTTCTGGGTGGCAGTGAT-3’). The internal control GAPDH was utilized to normalize all amplifications. All samples were assayed in triplicate, and all data were examined employing the 2^-△△Ct^ technique.

# **CCK-8 assay**

The experiment involved seeding cells in 96-well plates and subsequent transfection with pcDNA3.1 and pcDNA3.1-CNNM4. Following transfection, a 10 μL solution of CCK-8 (Beyotime, Shanghai, China) was introduced for each well to assess cell viability at 0, 24, 48, and 72 h. The detection of absorbance was conducted utilizing a FilterMax F5 plate reader set at a wavelength of 450 nm.

# **Flow cytometry**

The study involved the seeding of cells in 6-well plates and subsequent transfection with pcDNA3.1 and pcDNA3.1-CNNM4 for 48h. The cells were harvested and subsequently resuspended in 75% ethanol for an overnight duration. The cells were stained using the Cell Cycle Analysis Kit (Beyotime, Shanghai, China) following the manufacturer's rules. The cell cycle was quantified using SA3800 flow cytometer (SONY, Japan) and Modfit LT 5.0 (Verity Software House, San Jose, CA, USA). The cells collected underwent staining employing an Annexin V-FITC apoptosis detection kit (Beyotime, Shanghai, China) and were subsequently analyzed through a flow cytometer (SONY, Japan) following the manufacturer's directions. The trials were conducted separately and in triplicate.

# **Wound healing assay**

Cells were plated in each well of a 6-well plate, followed by incubation until they achieved complete confluency. Subsequently, the cells were transfected with pcDNA3.1 and pcDNA3.1-CNNM4. Following that, a scratch was created by a pipette tip. The medium was substituted with a medium that did not contain serum. Following a 24h incubation period, the process of wound closure was observed and recorded through the capture of images. The experiments were performed independently in triplicate.


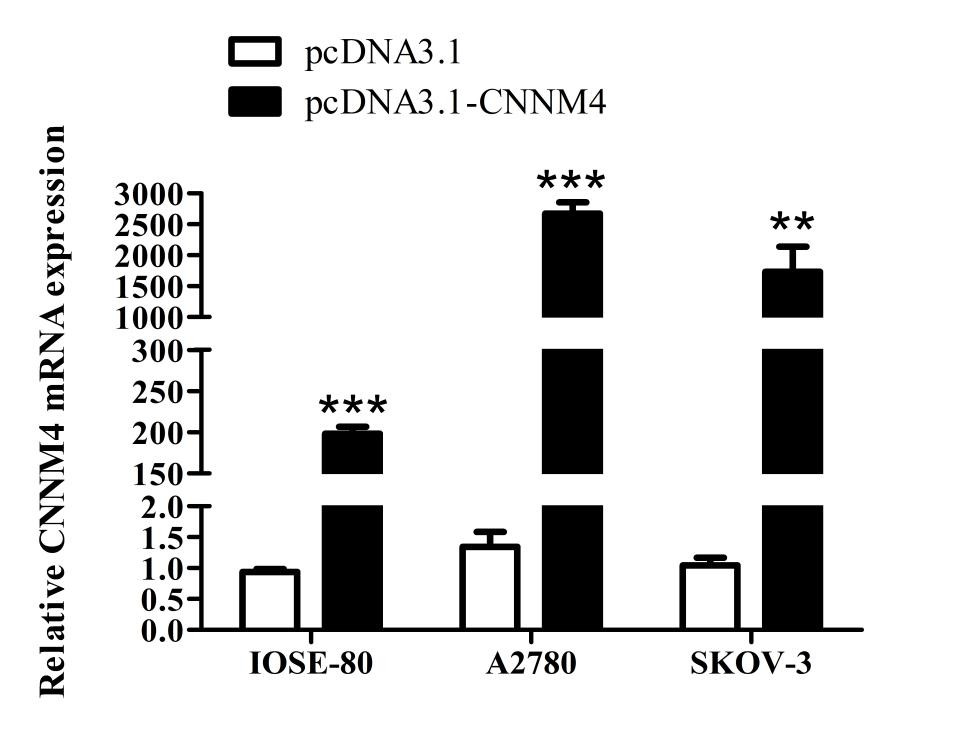


**Supplementary Figure 1**  qRT-PCR analysis of CNNM4 expression in IOSE-80, A2780 and SKOV-3 cells transfected with pcDNA3.1 and pcDNA3.1-CNNM4. The data were presented as the mean ± SEM obtained from three separate and independent experiments. “**” indicates P < 0.01, “***” indicates P < 0.001
